# An unusual cause of Cullen sign: blunt trauma and ruptured hemorrhagic pancreatic pseudocyst

**DOI:** 10.11604/pamj.2025.52.78.49326

**Published:** 2025-10-17

**Authors:** Chelsea Rui-Yan Chuah, Yoen Young Chuah

**Affiliations:** 1 Chung-Hwa Bilingual Elementary School of Arts, Kaohsiung, Taiwan,; 2 Department of Internal Medicine, Division of Gastroenterology and Hepatology, Ping Tung Christian Hospital, Ping Tung, Taiwan,; 3 Department of Nursing, Meiho University, Ping Tung, Taiwan

**Keywords:** Cullen sign, blunt trauma, pancreatic pseudocyst

## Image in medicine

A 48-year-old man with a history of alcohol-related acute pancreatitis presented with sudden, severe abdominal pain radiating to the back. He reported drinking six cans of beer daily (10.7 UK alcohol units) for over 20 years. Nineteen days earlier, he sustained blunt trauma to his left flank while lifting a heavy generator. Abdominal computed tomography (CT) at another hospital revealed a hemorrhagic pancreatic cyst, and he was transferred to our institution. Laboratory tests revealed normocytic anaemia with normal amylase, lipase, and carbohydrate antigen 19-9 levels. On day three of hospitalisation, his haemoglobin dropped from 14 g/dL to 9.6 g/dL, and a periumbilical ecchymosis appeared (Panel A). Repeat CT demonstrated rupture of the hemorrhagic pancreatic cyst with hemoperitoneum (Panel B). Diagnostic paracentesis confirmed bloody ascitic fluid (SAAG = 0.4). He was managed conservatively with blood transfusion and tranexamic acid, and was discharged after five days in stable condition. The periumbilical ecchymosis represents the Cullen sign, a rare clinical finding first described in 1918, caused by blood tracking from the peritoneal cavity to the umbilicus via the falciform ligament. Although classically associated with hemorrhagic pancreatitis, the Cullen sign may also be seen in ruptured ectopic pregnancy, ruptured abdominal aortic aneurysm, and hepatocellular carcinoma rupture. In this patient, the Cullen sign resulted from rupture of a hemorrhagic pancreatic pseudocyst, likely precipitated by previous trauma.

**Figure 1:**
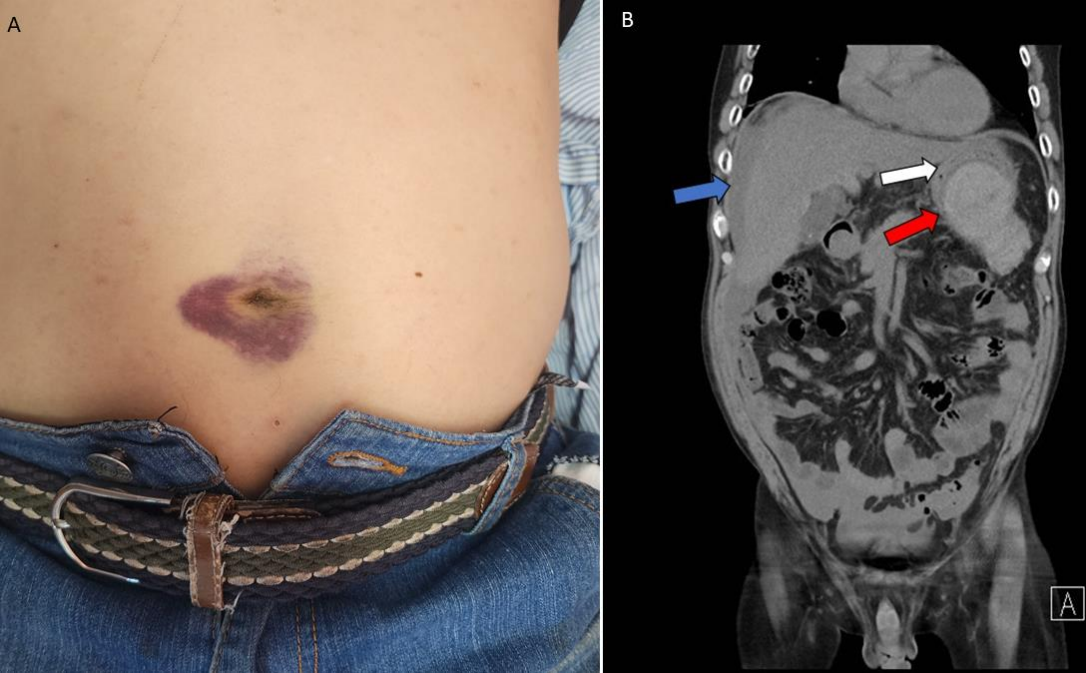
A) periumbilical ecchymosis consistent with Cullen sign; B) contrast-enhanced abdominal CT showing a hemorrhagic pancreatic cyst (red arrow) with associated hemoperitoneum (blue arrow) and compression of the stomach (white arrow)

